# Economic and emotional impact of COVID-19 pandemic on phoniatricians’ practice in Egypt

**DOI:** 10.1186/s43163-022-00217-x

**Published:** 2022-02-14

**Authors:** Aisha Fawzy Abdel Hady Ibrahim, Ayatallah Raouf Sheikhany

**Affiliations:** Phoniatric Unit, ENT Department, Faculty of Medicine, Cairo University, El Haram Street, Giza, 12511 Egypt

**Keywords:** COVID-19 pandemic, Phoniatricians, Egyptian community, Economic and emotional aspects, Cairo

## Abstract

**Background:**

COVID-19 is not only a health crisis; it has the potential to create devastating social, as well as economic crises. Health care practitioners are the category with the highest diffusion of the contagion. The aim was to determine the economic and emotional impact of the COVID-19 pandemic on phoniatricians in Egypt in an attempt to analyze this data to determine the magnitude of this effect and if it is age and/or location specific.

**Subjects and methods:**

An online structured Google-free form survey composed of 49 questions was created and sent online to phoniatricians all over Egypt. The survey was divided into three sections about demographic, economical then emotional-related questions. The studied group was further subdivided into 2 groups according to age and location for comparison purposes.

**Results:**

82.5% of phoniatricians confirmed that the pandemic had a lot of negative impact on their practice. About 37.5% reduced their practices to urgent procedures and the same percentage closed their practice. The expenses of 56.3% exceeded their income. 91.2% had negative feelings when thinking about the pandemic. The economic problems have affected 71.3% of the participants emotionally. The pandemic had comparable negative impact on the economic and emotional aspects of both age groups. However, the younger age group suffered more from getting infected, and they thought more about career shift and was the group that needed psychological support during the pandemic. Outside Cairo organizations succeeded in providing the personal protective equipment to the phoniatricians in comparison to Cairo.

**Conclusion:**

COVID-19 had a negative effect on the economical and emotional aspects of Egyptian phoniatricians’ lives. The pandemic economic burden was related to quarantine period, heath problems and getting infected, family requirements, and purchasing the protective equipment. The emotional burden was related most to the pandemic hazards and financial effect on the clients and chance of losing job. Few effects were age- and location-specific.

**Supplementary Information:**

The online version contains supplementary material available at 10.1186/s43163-022-00217-x.

## Background

The COVID-19 pandemic is part of the worldwide pandemic of coronavirus disease. The highly contagious disease caused by (SARS-CoV-2) started in Wuhan City in China in December 2019 and spread all over the world. The virus was confirmed to have reached Egypt on the 14th of February 2020 by the announcement of Egypt’s health ministry [1, 2]. In Egypt, by the beginning of April 2020, there were over 800 confirmed cases, with more than 50 fatalities, and a rapid tendency towards increase. In October 2020, the total number of reported cases was 107555. And the reported death rate rose to 6266 [3, 4]. COVID-19 has caused a global health emergency. Strong infection control measures and social distancing are the primary interventions to minimize the spread of the virus in both health care settings and the community till the emergence of proven treatments or vaccinations [5].

COVID-19 pandemic has caused numerous problems in people’s lives all around the world [6]. COVID-19 is not only a health crisis; it has the potential to create devastating social, as well as economic crises. Health care practitioners are the category with the highest diffusion of the contagion. In addition, the disease has caused many problems for healthcare providers and several occupations because of its high transmission rate. In Egypt, the Egyptian medical syndicate reported nearly 600 deaths of physicians in various medical fields till now and the number is increasing day after day. Among healthcare providers are the ear, nose, and throat (ENT) surgeons and phoniatricians as an ENT subspecialty who are at increased risk. As a poignant example, the first documented death of a physician was that of an ENT surgeon in China in January 2020 [7].

Physicians in medical fields and in private practices have been affected economically and emotionally by the outbreak of COVID-19 pandemic. Phoniatrics is a medical specialty dealing with voice, speech, language, hearing, and swallowing disorders. So phoniatricians are at risk because of the following factors: (1) exposure to aerosol generation in a procedure such as nasolaryngescopy; (2) flexible endoscopic evaluation of swallowing; (3) office-based practices such as vocal fold augmentation and in feeding; (4) oral motor evaluation and training as a result of presence of the virus in the nasal and pharyngeal cavities of infected individuals; (5) during head and neck examination, assessments, and interventions carried out in the upper airway and food passages; and (6) also in speech and language stimulation therapies, phoniatricians are dealing with both parents and children. It is now known that some asymptomatic individuals have been found to harbor active viral infection and to be potentially infectious to the same degree and with the same viral load as symptomatic individuals [8, 9].

So after the partial reopening in Egypt in July 2020, phoniatricians had to follow clinic- or hospital-related guidelines to keep themselves and their patients safe. Among the guidelines is using personal protective equipment such as masks, face, and eye protection; caps; and water disposable gowns provided by themselves or through their organizations. They have to reduce number of direct face to face exposure to patients so they tried the telehealth method in provision of history taking and some voice, speech, and language therapy. Being in such dilemma of keeping themselves safe, and keeping the balance between their incomes and expenses, they faced emotional problems and fear about their clinical future.

So this raised the utmost interest in studying to which degree the COVID-19 pandemic left its print on the practice of the phoniatricians in Egypt and the reflection it has on their economic and emotional lives.

The aim of this study was to determine the economic and emotional impact of the COVID-19 pandemic on phoniatricians in Egypt in an attempt to analyze this data to determine the magnitude of this effect with a second intention to determine if the effect is age- and/or location-specific.

## Methods

### Study design and population

A cross-sectional survey was created using the free-access Google form application, and the link to the online survey was sent using personal contact through What’s App to phoniatricians registered in the Egyptian Society of phoniatricians and logopedics (ESPL). Some of the ideas of the questionnaire were designed in light of a questionnaire carried out on dental practitioners in Italy [10]. The potential cultural factors around the phoniatric practice were taken into account while designing the current survey. All the questions were required to be answered, and only one answer was allowed to be chosen. Data collection took place in the period from the 11th of July to the 30th of July 2020 in the time of partial reopening.

The study was conducted on 80 Egyptian phoniatricians. The online structured survey composed of 49 questions was sent to phoniatricians allover Egypt in order to investigate how the COVID-19 pandemic has affected the economic and emotional lives of them. All the received responses were included in the study.

The sample size was calculated using the following equation: based on the primary objective and considering the following data, the power of the study is 90% and a margin of error is 8%.

$$\mathrm{Sample}\kern0.17em \mathrm{size}=\frac{{\left(\mathrm{z}\hbox{-} \mathrm{score}\right)}^{\wedge }2\times \mathrm{StdDev}\times \left(1-\mathrm{StdDev}\right)=75}{\mathrm{Confidence} \operatorname {int}{\mathrm{erval}}^{\wedge }2}$$

### Study tool

The survey was created and conducted in English as all the physicians, and the participants are from the medical field. English is their second language, and the language used in the daily medical practice. An online survey portal, Google form was created, and participants were invited to complete and submit the form. It was sent to phoniatricians trying to cover most of the regions in Egypt such as the two big governorates Cairo and Alexandria, in addition to Delta and Upper Egypt and other governorates. This study was approved by the Ethics Committee of Ear, Nose, and Throat Department, Cairo University. Respondent’s anonymity and confidentiality were ensured. The submission of the answered survey was considered as consent to participate in the study.

The 49 questions in the structured survey were divided into three sections:

- ***Section 1*** included 8 questions aimed at gathering demographic data; age, gender, marital status, family size, length of professional experience, location of practice, and type of professional setting in addition to history of emotional problems.
- ***Section 2*** was composed of 30 questions assessing the economic aspect of the phoniatricians by asking about the weekly working hours before and during the pandemic, degree of negative effect of pandemic on their practice, whether closure or reduction occurred as a result of the pandemic, the relation between their incomes and expenses, the cause of increased financial burden of phoniatricians during the pandemic, the availability and the price of personal protective equipment and how they are provided and whether the availability affected the clinical practice, the time of the closure or practice reduction, whether the patients understand the motives beyond the closure or practice reduction, the way consultations for urgent conditions conducted, main source of financial income, thinking about plan b, c, career shift, whether the phoniatricians regained their full practice after reopening, the used measures of ensuring safety, whether it is likely to get infected during the phoniatric practice, whether they got infected, place of remediation, how they covered their financial issues, knowing of colleague who got infected, and if it affected their decision to continue practice, degree the consequences affected them and in their opinion; to which degree the pandemic would continue to affect them, the main cause of financial burden, and how they spend their time during quarantine.
- ***Section 3*** was composed of 11 questions investigating the impact of the COVID-19 outbreak on the emotional aspect of the phoniatricians including the degree of worry from getting infection, their feeling when thinking about the pandemic, whether their stress level increased, whether they are affected emotionally, or other relation with their colleagues at work affected, or their productivity at work affected or their relations with their family and friends affected as a result of their professional and economic problems, in addition to whether they need medications for anxiety or depression, if they need psychological support, degree of worry about their future, and what worries them the most (See Additional file 1).

### Statistical analysis

It was done using IBM SPSS® Statistics version 22 (IBM® Corp., Armonk, NY, USA). Qualitative data were expressed as frequency and percentage. Pearson’s chi-square test or Fisher’s exact test was used to examine the relation between qualitative variables. All tests were two-tailed. A *p*-value < 0.05 was considered significant.

## Results

Table 1 shows the socio-demographic characteristics of the studied participants. Nearly half (55%) of the participants were between 31 and 40 years old. Three quarters (75%) of them were females. More than half of them (57.5%) were married and have children. Nearly 70% of them have a family size of 4 or more. The highest percentage (31.3%) of the participants had a professional experience of 5 to 10 years. More than half of the participants (56.3%) had their location of practice in Cairo. Around 51.2% of the participants had both public and private practices. Around 33.7% had a history of emotional problems.

**Table 1:** The percentages of the socio-demographic data of the studied group

Section I: Socio-demographic data	Choices	Percentages %
Age	▪ 20–30	13.8
	▪ 31–40	55
	▪ 41–50	16.3
	▪ Above 50	15
Sex	▪ Male	25
	▪ Female	75
Marital status	▪ Single	12.5
	▪ Married	30
	▪ Married & have children	57.5
Family size	▪ 2	8.8
	▪ 3	21.3
	▪ 4	36.3
	▪ More than 4	33.8
Length of professional experience	▪ Less than 5 years	13.8
	▪ 5–10	31.3
	▪ 11–15	20
	▪ 16–20	16.3
	▪ More than 20	18.8
Location of practice	▪ Cairo	56.3
	▪ Alexandria	3.8
	▪ Delta	22.5
	▪ Upper Egypt	12.5
	▪ Other	5
Professional setting	▪ Partner/owner of a private clinic.	8.8
	▪ Employed in private practice	8.8
	▪ Employed in public structure	31.3
	▪ Have public & private practice	51.2
History of any emotional problem	▪ No	66.3
	▪ Anxiety	17.5
	▪ Fear	2.5
	▪ Depression	11.3
	▪ Others	2.5

Table 2 presents the following collected data about the economical aspect of the studied group of phoniatricians during the pandemic of COVID-19.

**Table 2:** The percentages of the collected data about the economical aspect of the studied group of phoniatricians during the pandemic of COVID-19

Section II: Economical section data	Choices	Percentages %
Q9 Weekly average working time before the pandemic (Mid- March till present time)	▪ More than 25 h	45
	▪ 20–25 h	22.5
	▪ 10–19 h	17.5
	▪ Less than 10 h	13.8
	▪ Not working	1.3
Q10 Weekly average working time during the pandemic (Mid-March till present time)	▪ More than 25 h	1.3
	▪ 20–25 h	3.8
	▪ 10–19 h	17.5
	▪ Less than 10 h	40
	▪ Not working	37.5
Q11 The likelihood of the negative effect of the pandemic on practice	▪ A lot	82.5
	▪ a little	16.3
	▪ Not at all	1.3
Q12 Continuation of practice during COVID-19 pandemic	▪ Continued the same	3.8
	▪ Closed	37.5
	▪ Reduced to 50% of its power	21.3
	▪ Reduced to urgent procedures/consultations	37.5
Q13 The relation between expenses & income during COVID-19 pandemic	▪ I keep the balance between my income & expenses	41.3
	▪ My expenses exceed my income	56.3
	▪ My income exceeds my expenses	2.5
Q14 The cause of increasing of expenses burden in the COVID-19 pandemic	▪ Protective personal equipment financial burden	10
	▪ Internet usage	6.3
	▪ Family issues	8.8
	▪ Decreased income	17.5
	▪ All of the above	50
	▪ None of the above	7.5
Q15 Availability of PPEs	▪ Available	73.8
	▪ Not available	26.3
Q16 Providing PPEs	▪ Provided by your organization	27.5
	▪ You have to buy them at your expenses	72.5
Q17 The effect of the availability of the PPEs on clinical practice	▪ Yes	51.2
	▪ No	10
	▪ Sometimes	38.8
Q18 The price of the personal protective equipment during the pandemic	▪ Is the same	7.5
	▪ Has increased to a large extent	92.5
Q19 PPEs used before the pandemic	▪ Gloves, masks, white coats	91.3
	▪ Gloves, masks, gowns	2.5
	▪ Gloves, masks, face shield & disposable gowns	6.3
Q20 The time of complete or partial closure of work	▪ Just when the danger of the pandemic was felt	50
	▪ After the national quarantine was declared	50
Q21 Patients understanding of the motives beyond the closure or reduction	▪ Yes	58.8
	▪ No	1.3
	▪ Not all of them	40
Q22 Way of conduction of consultations for urgent conditions	▪ Through telephone	20
	▪ Online consultation	18.8
	▪ Face to face	11.3
	▪ More than one of the above	31.3
	▪ All of the above	18.8
Q23 The main source of financial income during the COVID-19 pandemic	▪ Online sessions	3.8
	▪ Governmental salary	65
	▪ Private Practice	5
	▪ Other practices than Phoniatrics	7.5
	▪ More than one of the above	18.8
Q24 Thinking about plan b & c to cover finances during the pandemic	▪ Yes	32.5
	▪ No	35
	▪ Did not find any idea to help	32.5
Q25 Thinking about career shift	▪ Yes	70
	▪ No	30
Q26 Regaining of full practice after the national reopening in case of partial or complete closure of work	▪ Yes	86.3
	▪ No	13.8
Q27 Safety measures from infection	▪ Body temperature measurement	
	▪ Asking about symptoms	
	▪ Usage of disinfectants	3.8
	▪ Reducing number of cases	
	▪ Providing cases with masks & gloves	
	▪ More than one of the above	53.8
	▪ All of the above	42.5
	▪ None of the above	
Q28 The likelihood of getting infection during phoniatric practice	▪ Yes	77.5
	▪ No	2.5
	▪ May be	20
Q29 Getting infected with coronavirus	▪ Yes, confirmed case	1.3
	▪ Suspected case	16.3
	▪ No	82.5
Q30 Place of remediation from COVID-19	▪ Home (isolation)	18.8
	▪ Hospital	
	▪ Did not need	81.3
Q31 Covering the financial issues	▪ Medical insurance	7.5
	▪ Own budget	92.5
Q32 Knowing colleagues in the field of Phoniatrics who were COVID-19 positive	▪ Yes	13.8
	▪ No	86.3
Q33 Emotional effect of the pandemic	▪ Scared	12.5
	▪ Frustrated	7.5
	▪ Anxious	38.8
	▪ Concerned	27.5
	▪ None of the above	13.8
Q34 The pandemic effect on decision about continuing practice during the pandemic	▪ Yes	32.5
	▪ No	67.5
Q35 The degree of the effect of the consequences of the COVID-19 pandemic	▪ Major degree	46.3
	▪ Moderate degree	48.8
	▪ Minor degree	5
Q36 The degree the pandemic will continue to affect daily practice	▪ Major degree	35
	▪ Moderate degree	57.5
	▪ Minor degree	7.5
Q37 The burden causes during the pandemic	▪ Being in quarantine	1.3
	▪ Children care	6.3
	▪ Health & being sick with coronavirus	6.3
	▪ Financial issues	3.8
	▪ Uncertainty about job	3.8
	▪ Increasing conflicts with people around	--
	▪ More than one of the above	62.5
	▪ All of the above	16
Q38 Spending time during quarantine	▪ The same routine as before	1.3
	▪ Care of family	18.8
	▪ Developing professionally	1.3
	▪ Part time work	
	▪ Reading & searching about the corona news	1.3
	▪ More than one of the above	77.5

45% of the studied group had more than 25 h as an average of their weekly work before the pandemic while 37.5% of them were not working during the pandemic. The majority (82.5%) of them confirmed that the pandemic had a lot of negative effect on their practice. About 37.5% reduced their practices to urgent procedures and consultation, and the same percentage closed their practice. The expenses of more than half of them (56.3%) exceeded their income. Half of the studied group referred to the increase in their expenses’ burden collectively to a lot of factors such as personal protective equipment financial burden, Internet usage, family issues, and decrease of the income. 73.8% found that PPEs were available while 72.5% of them pointed out that one should buy them at his/her expenses. 51.2% admitted that the availability of PPEs affected their clinical practice. 92.5% confirmed that the price of PPEs have increased to a large extent during the pandemic. 91.3% tended to use only gloves, masks, and white coats before the pandemic. The participants were divided equally to those who closed partially or completely just when the danger of the pandemic was felt and the second half closed after national quarantine was declared.

More than half of the studied group, 58.8% found that their patients understand the motives beyond the closure or reduction of work. 31.3% tended to use more than one method to conduct consultations for urgent conditions either through telephone, online consultation, or face to face. The governmental salary was the main source of financial income during the pandemic in case of 65% of the studied group. 32.5% thought about plan b and c to cover their finances during the pandemic. 70% thought about career shift. 86% regained their practice after the reopening either partially or completely. 42.5% used all the protective measures.

The opinion of 77.5% of participants that it is likely to get infected during phoniatric practice. 16.3% were suspected cases for coronavirus infection, and 1.3% were confirmed cases. 18.8% received remediation through home isolation. 92.5% covered their financial issues during the period of their infection with their own budget. 13.8% knew that some of their colleagues in the phoniatric field who got infected. 38.8% of them got anxious, and 27.5% got concerned that consequently affected the decision of 32.5% of the studied group to continue their practice during the COVID-19 pandemic. The consequences of COVID-19 affected 48.8% and 46.3% of participants to moderate and major degree, respectively. 57.5% of the studied group thought that the pandemic would continue to affect their practice to moderate degree. 62.5% found more than one factor of burden during the pandemic. 77.5% spent their time during quarantine in more than one of the following; the same routine as before, family care, developing professionally, part time work, reading, and searching about corona news.

Table 3 presents the following collected data about emotional aspect of the studied group of phoniatricians during the pandemic of COVID-19.

**Table 3:** The percentages of the collected data about the emotional aspect of the studied group of phoniatricians during the pandemic of COVID-19

Section III: Emotional section data	Choices	Percentages
Q39 Degree of worry from getting infection/reinfection during practice	▪ A lot	65
	▪ A little	33.8
	▪ Not at all	1.3
Q40 Type of feeling when thinking about COVID-19 pandemic	▪ Sad	20
	▪ Angry	3.8
	▪ Concerned	15
	▪ Scared	18.8
	▪ Anxious	33.8
	▪ None of the above	8.8
Q41 The likelihood of increasing stress level as a result of the pandemic	▪ To a great extent	36.3
	▪ To a moderate extent	41.3
	▪ To a little extent	22.5
Q42 The degree of the emotional effect of professional & economical problem	▪ To a great extent	30
	▪ To a moderate extent	41.3
	▪ To a little extent	28.7
Q43 The likelihood of the effect of professional & economical problem on relations with colleagues at work	▪ Yes	82.5
	▪ No	17.5
Q44 The likelihood of professional & economical problem on productivity at work	▪ Yes	60
	▪ No	40
Q45 The Likelihood of effect of professional & economical problem on relations with family and friends	▪ Yes	66.3
	▪ No	33.8
Q46 The need to take medications for anxiety or depression	▪ Yes	6.3
	▪ No	93.8
Q47 The need of psychological support	▪ Yes	23.8
	▪ No	76.3
Q48 Degree of worry about professional future	▪ A lot	36.3
	▪ A little	47.5
	▪ Not at all	16.3
Q49 Most causes of worry	▪ The pandemic	60
	▪ Corona will affect the clients financially & economically	16.3
	▪ Chance of losing job or being laid off by employer	1.3
	▪ Other	22.5

65% of the participants were worried a lot from getting infection/reinfection during the pandemic of COVID-19. 91.2% had negative feelings when thinking about COVID-19 Pandemic. 36.3% of the participants’ stress level had increased respectively to a great extent.

The professional and economic problems affected 30% of the participants emotionally to a major degree. The professional and economic problems affected 82.5% of the participants in their relation with their colleagues in work, also affected 60% of the participants in their productivity and 66.3% of the participants in their relation with their family and friends.

Only 6.3% of the participants needed to take medications for anxiety or depression. Only 23.8% needed psychological support. 63.3% were worried about their professional future. The pandemic was the issue that worries 60% of the participants followed by the effect of the corona on the clients’ finances and economy.

Table 4 presents the comparison between the two age groups as regards their responses to some economic aspects. Significant difference between the two age groups were only seen in thinking about career shift and getting infected with corona virus as the younger age group had the higher percentage.

**Table 4:** Comparison between younger and older age groups regarding the percentage of their responses to some economic aspects

		≤ 40 years	>40 years	*P* value
Q9 Working hours before pandemic	>25	45.5	44	0.580
	20–25h	21.8	24	
	10–19h	14.5	24	
	<10 h+ Not working	18.2	8	
Q10 Working hours during pandemic	≥10	21.8	24	0.474
	<10	36.4	48	
	Not working	41.8	28	
Q11 Negative effect on practice	A lot	83.6	80	0.692
	A little/not at all	16.4	20	
Q13 Relation between expenses & income	Balance+ income exceeds expenses	43.6	44	0.976
	Expenses exceed income	56.4	56	
Q23 Main source of financial income during pandemic	Governmental salary	61.8	72	No *p* value due to small number of cases within the subgroups
	Online sessions	5.5	0	
	Private practice	7.3	0	
	Other practices than phoniatrics	7.3	8	
	More than one of the above	18.2	20	
Q24 Thinking about Plan B, C	Yes	40	16	0.105
	No	30.9	44	
	Did not find any idea to help	29.1	40	
Q25 Thinking about career shift	Yes	38.2	12	0.018*
Q29 Infection with coronavirus	Confirmed & suspected cases	25.5	0	0.005*

*denotes significant *p* value

Specific considerations were found during the comparison among the different 4 age groups regarding the following: 63.6% of the youngest age group (20–30 years) worked above 25 h/week before the pandemic while during the pandemic, 45.5% of the same age group was not working and 36.4% worked between 10 and 19 h. The majority which were 43.2%, 46.2%, and 50% of the 31–40 years, 41–50 years, and above 50 years age group, respectively, worked less than 10 h.

In response to a question about the negative effect of the pandemic on practice, a percentage ranged between 54.5% of the (20–30 years group) to 100% of above 50 years age group found that the pandemic had a lot of negative effect on their practice.

Regarding the response to the relation between expenses and income during the COVID-19 pandemic, the highest percentage (63.6%) of 20–30 years of age group was trying to keep the balance between their income and expenses while a percentage of 61.5% of (31–40 years) age group stated that their expenses exceeded their income.

As regards the main source of financial income, the highest percentage in the four age groups went for the governmental salary ranging between 54.5% of the 20–30 years of age group to 84.6% in the 41–50 years of age group.

The higher two age groups, those of 41–50 and above 50 years, did not depend at all on “online sessions or private practice” during the pandemic. The highest percentage for online sessions (18.2%) was found in the youngest age group (20–30 years).

The highest percentage who thought about career shift, 40.9% of the 31–40 years of age group and a percentage ranging from 27.3 to 41.7% of 31–40 and above 50 years, respectively, did not find any idea to help them during the pandemic. As high as 63.6% in the youngest age group and as low as 8.3% of the above 50 age group thought about career shift.

Regarding infection with coronavirus, suspected cases were found in the youngest age groups with percentages of 45.5% and 18.2% in the 20–30 and 31–40 age groups, respectively.

Table 5 presents the comparison between the two age groups as regards their responses to some emotional aspects. The only significant difference seen was in the worry to get infected or re-infected as the younger age group got the higher percentage.

**Table 5:** Comparison between younger and older age groups regarding the percentage of their responses to some emotional aspects

		≤ 40 years	>40 years	*P* value
Q35 Degree of pandemic effect	Major	50.9	36	0.215
	Moderate+ Minor	49.1	64	
Q36 Degree that the pandemic will continue to have effect	Major	40	24	0.251
	Moderate	50.9	72	
	Minor	9.1	4	
Q39 Degree of worry from getting infection/reinfection	A lot	72.7	48	0.032*
	A little/not at all	27.3	52	
Q41 Degree of increased stress level due to pandemic	Great	41.8	24	0.172
	Moderate+ little	58.2	76	
Q46 Need to take medications from anxiety or depression	No	94.5	92	0.645
	Yes	5.5	8	
Q47 Need for psychological support	Yes	29.1	12	0.096
Q48 Degree of worry about professional future	A lot	40	28	0.539
	A little	43.6	56	
	Not at all	16.4	16	
Q49 Causes beyond worry	Pandemic	60	60	0.322
	Corona will affect clients financially	12.7	24	
	Losing job +others	27.3	16	

*denotes significant *p* value

Special consideration when comparing the four age groups regarding some questions related to emotional aspect;

46.3% and 48.8% of the participants found that the consequences of the pandemic affected them to major and moderate degrees. Also 35% and 57.5% thought it would continue to affect their daily practices to major and moderate degrees.

65% were worried from getting infection or reinfection from coronavirus with the highest percentages; 77.3% and 54.5% found in the youngest two groups (31–40, 20–30) years old, respectively.

36.3% and 41.3% of the participants had their stress level increased to a great and moderate extent as a result of the pandemic. The highest percentage found was about 72.7% in the youngest age group (20–30 years). 6.3% of the participants needed to take medications for anxiety or depression, and the highest percentage found was about 8.3% of the highest age group (above 50 years). 23.8% needed psychological support and the highest percentages were found in the youngest two age groups about 45.5% followed by 25% in the two age groups of (20–30, 31–40 years), respectively.

36.3% were worried a lot about their professional future; with the highest percentage (63.6%) of the age 20–30 followed by the percentage of 34.1% of the age (31–40). 47.5% of the participants were worried a little about their professional future, with the highest percentage, 58.3% of the above 50 years of age group followed by 53.8% of the 41–50 years of age group*.*

The pandemic was what worries 60% of the participants a lot with a range from 50% in the above 50 group to 69.2% in the 41–50 years of age group.

Table 6 presents a comparison between the percentages of the responses of phonitricians located in Cairo and outside Cairo regarding some economic aspects. It showed that the only significant difference found was providing the personal protective equipment as phoniatricians located in Cairo had to provide the equipment at their expenses more than that of phonitricians located outside Cairo whose equipment were provided mainly through their organizations.

**Table 6:** Comparison between the percentages of the responses of phoniatricians located in Cairo and outside Cairo regarding some economic aspect

		Cairo	Outside Cairo	*P* value
Q15 Availability of personal protective equipment	Available	75.6	71.4	0.677
Q16 PPEs are provided by	Organization	17.8	40	0.027*
	At your expenses	82.2	60	
Q17 Availability of PPEs affecting practice	Yes	48.9	54.3	0.344
	No	6.7	14.3	
	Sometimes	44.4	31.4	
Q18 Price of PPEs during pandemic	Increased	93.3	91.4	1
Q21 Patients’ understanding of motives beyond closure	Yes	57.8	60	0.841
	No+ not all of them	42.2	40	

*denotes significant *p* value

## Discussion

The devastating effect of COVID-19 pandemic extended to the medical field practitioners, their private practices, and their personal lives. This survey is exclusively directed to investigate the impact of COVID-19 pandemic on both the economic and consequently emotional lives of phoniatricians in Egypt.

Data showed in Table 1 presented the demographics of participants in this study; 80 phoniatricians filled in the survey. Interestingly, the majority of the participants were from the younger age group and were females. More than half of participants were married with children. This goes with the age and gender demographic data earned from ESPL as most of the phoniatricians in Egypt are females. Younger age groups tend to be more social-media active, and thus, younger participants were more likely to answer the survey. This helped to have an idea about the economic and emotional circumstances of this young adulthood group who have family responsibilities during the pandemic of COVID-19. The majority have a professional experience ranging from 5 to 20 years. The data covered the three main areas in Egypt, Cairo, the Egypt’s capital, Lower Egypt (Delta), and Upper Egypt. Nearly half of the participants had both public and private practices which is the usual scenario for practice here in the country. The other half of the participants had only one professional setting either public or private practice. Nearly one third of the participants had a history of emotional problems and the most prevalent problem was anxiety.

On analyzing the data presented in Table 2; the spread of the pandemic cut the working hours to less than10 h weekly or not working at all. This goes in agreement with a study of Chadd et al. [11] that pointed to reduction of caseloads for speech and language therapists (SLTs) in the UK, and they attributed this in part to be caused by fewer new referrals for the assessment and treatment of speech, language, and communication disorders in addition to the effect of SLTs being unable to provide intervention to individuals on existing caseloads following the closure of settings during lockdown, and patients opting not to access services at this time. In the current study, the majority of participants admitted that the pandemic had a lot of negative impact on their practices as only 3.8% continued their practices the same as before while the majority of the participants closed, reduced their work to urgent procedures or consultations, or reduced to 50% of its power. This could be explained by their response to the Egyptian government that called for the quarantine by the mid of March 2020. Various occupations and service-providing centers were temporarily closed down due to the necessity of quarantine during the COVID-19 outbreak [12]. This was especially true for services that require a close distance between the service receiver and the service provider [13]. Phoniatricians usually require face-to-face communication with their patients to provide assessment and treatment services. As a result, the provision of services in this field had seriously been interrupted so as to observe the necessity of quarantine and social distancing.

The decreased working hours, and the reduction or closure of practices reflected negatively on the expenses of participants as more than half of the participants had their expenses increased compared to their incomes and about 41.3% had to reduce their expenses in order to keep the balance between the income and expenses. This goes with the condition caused by the pandemic all over the world as coronavirus led to a great extent of economic and financial instability due to reduction in clinical work times [14]. This is also in agreement with a study done on the Egyptian physicians by Abdel Hafiz et al. [15] whose results indicated that the economic burden during the epidemic was a significant concern for their respondents.

Half of the participants attributed the increased burden of their expenses during the pandemic time collectively to a number of factors including decreased income, protective personal equipment financial burden, family issues, and the Internet usage. This was a logical explanation as the income was extensively affected due to the reduced number of cases attending their practice causing a significant income reduction, especially in small private practices. In order to continue work even for limited times, appropriate protection and the use of disinfection along with the use of disposable instruments were mandatory and of prime importance with additional financial burden [16]. In addition to the increased price of the PPEs as confirmed by the participants as will be discussed later. The time of the quarantine was spent at home with increased expenses for the family requirements with an increase of the internet usage to follow the corona news, the educational purposes for children, and also for entertainment.

The previous facts were reported in the current study as 42.5% of the participants attributed the financial burden to one factor only of those mentioned before with the highest percentage goes to the decreased income then to the PPEs financial burden. In fact, 73.8% of the participants recorded the unavailability of PPEs in their work places and the majority (72.5%) of the participants reported that they had to buy them at their own expenses and only 27.5% of the participants reported that the PPEs were provided by their organizations. So the PPEs’ financial burdens were one of the factors that contributed to a great extent to the increased financial and economic burden of the practitioners in the phoniatric field. The majority (92.5%) of phoniatricians reported that the price of the PPEs has increased to a great extent during the time of the pandemic. In addition, the availability of the PPEs affected the clinical practice of about more than half of the participants (51.2%). The PPEs that 91.3% of the participants depended on before the pandemic were masks, gloves, and white coats, these were not enough to ensure safety of the practitioners and the phoniatricians had to add other PPEs such as face shields, eye goggles, water disposable gowns following the national standards agreed upon in Egypt, and international ones followed all over the world. These equipment were considered the minimum PPE required to use when examining or performing a potential aerosol generating procedure or operating [17, 18] and when dealing with patients (adults or children) with head and neck issues. It is now well known that COVID-19 can be easily transmitted in cases of close contact between people in case the necessary PPEs are not used [19, 20]. The results of the study of Abdel Hafiz et al. [15] on Egyptian Physicians declared the same as more than half of their participants were satisfied with the availability of PPE in their hospitals; many of them had to buy PPE out of their own pockets. Different hospitals tried to provide medical personnel with basic PPEs. However, extra protective measures like using face shield masks and N95 masks were needed during the pandemic. Physicians in their study had to pay for them and look for other sources to purchase these supplies.

Table 2 represented some of the questions addressing the behaviors of the phoniatricians in Egypt towards the COVID-19 pandemic. The participants were divided equally to those who initiated closing or reducing their work once they felt the danger of the pandemic before the national quarantine was declared while the other half reduced or closed their practice once the quarantine was imposed. The patients of more than half of the participants understood the motives beyond the closure while the others were not satisfied as the negative effects of children's disorder led to physical, psychological, social, and economic discomforts in those parents [21, 22]. The COVID-19 outbreak has probably caused other problems for these families, increasing the parents’ physical and psychological issues during the pandemic [12]. This negatively affected the quality of parental care and conducting speech-language exercises, and this is why the families of these children had typically double the burden of financial issues to treat their children’s problems [23]. Meanwhile, with the outbreak of COVID-19 disease and its negative economic effects, families were likely to be under more economic pressure [24]. Families of these children dealt with various psychological issues due to their children’s problems [22], which were intensified by their concerns regarding the treatment of their children’s speech language problems. Overall, it seems that COVID-19 outbreak had increased the problems and concerns in families with children who suffered from various communication disorders and inabilities. Moreover, the sudden onset of certain speech and language disorders (e.g., stuttering) in children could have increased parents’ concerns for immediate assessment of the child’s condition [25].

One or more of the alternative methods were used by the participants of the current study to conduct consultations for urgent conditions; telephone call “voice & video calls,” online “zoom application,” and face to face. The most commonly used were phone calls then online consultations, and the least was face to face. As the COVID-19 pandemic had a drastic effect on the activity of phoniatricians, they headed to other ways to present their services because of the risk of diagnostic delays and the drop in the progress of the cases that were in therapy and needed intensive rehabilitation. The measures of the lockdown made it difficult to perform scheduled and new examinations in a timely manner, causing the risk of diagnostic delays with severe impact on patients’ health. This goes with the finding of the study done by Chadd et al. [11] that pointed to adopting different methods of service delivery by their participants using a variety of technologies. The “switchover” to telehealth has been one of the widest reported changes to healthcare in this period.

The outbreak of COVID-19 and closing of most phoniatric centers deprived children who were in need of phoniatric services causing numerous problems for them and their families. It was therefore critical to find a solution to provide favorable and high-quality services to these children at times such as the COVID-19 outbreak, when the in-person provision of services was impossible [6]. All the previously mentioned highlighted the importance of providing consultations by other means. This was also a trial to balance the income of phoniatricians to compensate for the financial requirements and needs of themselves, their families, and their employees and assistants in the private practice. Phoniatricians mostly rely on communication through visual-auditory and perceptual aspects; telepractice could be a proper opportunity to provide care in this field. In addition, many health care problems, such as rehabilitation, can be solved through the telepractice protocols.

In the current study, 32.5% of participants thought about other plans and other sources of income. 70% of the participants thought about making a career shift. This could be considered subsequent consequences to the threat of the affected economic level and decreased income. This is also was reported by the Egyptian physicians who participated in the study of Hafiz et al. [15].

After the national reopening, the majority (86.3%) regained their full practice. In order to keep them and their patients safe, and because the majority of the participants (77.5%) were aware of the likelihood of getting infected during practicing, they reported to use all or some of the following measures “body temperature measurement, asking about symptoms, usage of disinfectants, reducing number of cases, and providing cases with masks and gloves.” Another important factor that was revealed in the current study and contributed to the financial burden of the participants is that 16.3% of them had already been suspected cases and only 1.3% was confirmed cases. Although none of them needed hospitalization but 18.8% of them needed home isolation. This is an important factor to add to their financial burden, as they covered their own expenses of recovery from COVID-19.

Being surrounded by news of colleagues in the phoniatric field who got infected with COVID-19 raised various kinds of negative emotions such as anxiety, getting concerned, scared and frustrated. The effect extended to about one third of participants in making the decision to continue practice during the pandemic. 93 to 95% of the participants confessed that the consequences of the pandemic have affected them in major and moderate degrees, and they thought the pandemic consequences would continue to affect their daily practice in the medical field as any other private and small practices in the country affected. This is also shown in the finding of the study of Hafiz et al. [15] as infection of a colleague with COVID-19 was significantly correlated with higher emotional exhaustion score for the participants in their study. They attributed this to that fears associated with the infection of colleagues exaggerate the professional stress and the feeling of being emotionally worn-out and drained.

Table 3 represented questions addressing the emotional aspect of the studied group under the circumstances of COVID-19 Pandemic. Two thirds of the studied group worried a lot from getting infected or reinfected during practice. This is because the practice of phoniatricians exposed them to being in contact with the children and their caregivers, even with strict measures, the children may be carriers without any apparent symptoms. Also a great extent of services provided required dealing with patients who have laryngeal and nasopharyngeal symptoms and performing different diagnostic interventions. This subjects the phoniatricians to hazards of infection or reinfection even with strict measures. Meanwhile, provision of phoniatricians’ services requires full communication between them and patients, especially children. Given the necessity of having face-to-face communication, observing the speech articulators of phoniatrician by children, and establishing eye contact, phoniatricians cannot practically use PPE and may even need to touch the child for some reasons or use special toys to communicate with the children, which severely increase the risk of disease transmission.

Nearly all the participants had different kinds of feelings when they thought about the pandemic. Among the most prevalent feelings were anxiety, sadness, and fear. The current study pointed to the causes beyond the negative emotional impact of the pandemic on the participants as the news about the pandemic and the health hazards raised the stress level of 75% of the participants to great and moderate extent. The deterioration of the economic aspect of subjects under study had negative impact on their emotional health to a great and moderate extent. The current study declared that this subsequently affected the relations of the participants with their colleagues at work and affected the productivity of the most of them. The findings of the current study are in agreement with Ahmed et al. [26] who conducted their study on more than 500 Egyptian participants; one fifth of them were health care providers. In their study, the participants exhibited a moderate level of fear of the disease. More health care workers than non-health care workers expected worse outcomes from the epidemic. Moreover, COVID-19 exerted a negative impact on the different aspects of life of the participants, which added to the fear regarding the pandemic. They related their results to the regular following of health information, increasing concern regarding news about infection and death rates, expectation of worse outcomes from the pandemic, negative impact on social and daily lives, decreased income, and increased risk at work. The negative emotional feelings like depression and anxiety were reported in other studies addressing the effect of implementing mass quarantine and/or social distancing as that happened in some countries like India that applied social distancing to prevent the spread of SARS. Applying such measures indicated that the situation had become severe and was liable to worsen in future with a sense of being trapped and perception of loss of control [27].

The current study showed that the negative effect of the economic and emotional aspects on the participants extended to their relations with family and friends in about 66.3% of them. The emotional difficulties the participants faced did not need intervention with medications, but some of them (23.8%) admitted the need for psychological support. Thirty-six percent of phoniatricians under study got worried a lot about their professional future. Variable causes were incorporated to raise the worry in participants under study; the pandemic itself, the corona that will affect the clients financially and economically and to lesser extent the chance of losing job or being laid off by the employer. In conclusion, the negative emotions experienced after coronavirus is related to not only the health hazards but also the deterioration of the economic level of both the practitioners and their clients. The findings in the current study shed light on the pandemic effect on the emotional lives of participants under study and the causes beyond this. This highlighted the need for professional psychological care and the access to reliable information for minimizing the mental health problems during quarantine.

As a trial to evaluate if the effect of the pandemic was age specific, comparison between two age groups (less than 40 and above 40) in some economic aspects was carried out as seen in Table 4. The current study showed that the two groups have comparable responses with no significant difference regarding their working hours before and after the pandemic. The highest percentages tended to work more than 25 h before the pandemic in both groups while the highest percentages tend to work less than 10 h or even stopped working during the pandemic in both groups.

The two groups reported that the pandemic had a lot of negative impact on their practice. The highest percentages in both groups had their expenses exceeding their income; they depended on their governmental salary as a main source of their income. However, the younger age group thought more than the older age group about making a career shift and that they had a higher percentage of coronavirus infection (confirmed and suspected cases) than the older age group. This result was expected since in the practice of phoniatrics here in Egypt, most of the Phoniatricians above 40 have a stable private work and it is common that they have a lot of assistants from younger phoniatricians or speech and language pathologists who work with them, so they are less subjected to direct exposure to cases or subjected but for a shorter time than the young phoniatricians who are trying to build in their career and get more experience in dealing with cases. This might explain the increased number of infected cases in younger age group compared to older ones and their thoughts about having a career shift.

Table 5 represents the comparison between younger and older age groups regarding the percentage of their responses to some emotional aspects. They showed comparable results with no significant difference regarding the degree of pandemic effect on them. The majority of both groups found that the pandemic would continue to affect them to major and moderate degrees. They both had an increase in their stress level. Both groups did not need to take medications. Both groups were concerned about their future due to many causes especially the pandemic itself which worried them a lot. However, the younger age group had higher percentage than the older age group as regards the worry to get infected or reinfected as the confirmed and suspected cases were already seen in higher percentage in the younger than the older age group as seen in Table 4. The younger age group showed higher percentage with borderline significance than the older age group in the need for psychological support.

In conclusion, the current study showed that the pandemic had a comparable negative impact on the economic and emotional aspects of both age groups for phoniatricians under study. However, the younger age group suffered more than the older age groups from getting infected and had more fear from getting reinfected, they thought more about career shift and was the group that needed psychological support against the effect of coronavirus pandemic. This result is partially in agreement with Rouse and Regan [28] who stated that speech and language therapists belonging to younger age groups, who have fewer years of clinical experience, are at highest risk of depression, anxiety, and stress during the COVID-19 pandemic. They attributed the emotional vulnerability of the younger age group to be negatively affected by the pandemic to that in their study, younger SLTs were singles with possibility of lack of people in their lives to provide significant social support in such isolating time in the pandemic. This is not in agreement with the case in this study as previously mentioned that most of the participants were married and had children. However, being younger in age and less experienced made them in doubt about their career as an effect of facing the “Unknown” of the pandemic and this goes with the results of the study of Hafiz et al. [15].

As a trial to evaluate if the effect of the pandemic is location specific, comparison between responses of phoniatricians under study from Cairo and outside Cairo was done in Table 6. Responses in both locations were comparable with no significant difference as regards availability of personal protective equipment; phoniatricians in both locations found that the availability of personal protective equipment affected their practice. The price of PPEs increased in both locations during the pandemic and in both locations, more than half of the phoniatricans’ responses found that the patients understand the motives beyond the closure. However, in the outside Cairo group, the organizations took the responsibility to provide PPEs to phoniatricians at their work more than in Cairo that showed inadequate or less support to provide PPEs. This might be explained by the pressure of the increased case load in Cairo being the capital government of Egypt. The finding of inadequate PPEs in Cairo hospitals is in agreement with a study done by Sayed et al. [29] whose study was conducted on more than 250 house officers in Cairo, they perceived the institutional preparedness to be unsatisfying as regards the availability of PPEs and the perceived training.

### Limitations of the study

This study was a trial to collect data about the phoniatricians’ economic and emotional status in the era of COVID-19. However, the study has some limitations that need to be addressed in future studies as follow-up of participants over time is needed. This will add a value about the changes in their lives while getting along with the pandemic. The sample size under the study was relatively small so doing the research on a larger scale is recommended. Future research studies evaluating the phoniatricians’ economic and emotional status in comparison to the wider public physicians may be of a significant value. This will add a lot to understand the degree of severity of the pandemic effect on the phoniatricians’ status. Validated and reliable-based tools for assessment of the emotional and psychological status of the phoniatricians are recommended in future studies. In addition, some items can be added to the questionnaire such as the inquiry about the exposure of the participants to adequate training to deal with such an outbreak either by their institutions or by their own and the inquiry if vaccination was one of the available suggested safety measures from infection.

## Conclusion

This study shed light on the pandemic effect on phoniatricians in the Egyptian community as regards both the economical and emotional aspects and the behaviors of phoniatricians towards the pandemic in addition to their trial to find other ways to provide services, taking into consideration safety measures for them and their patients. It also showed their thoughts about other plans or even career shift as in younger age group below 40. The governmental salary as a stable income source supported a lot those who work within the governmental institutions in their financial battle against corona. The current study highlighted that the younger age is the age of vulnerable phoniatricians susceptible to psychological distress so that these younger age group can be adequately supported. Outside Cairo organizations succeeded in providing PPEs to the phoniatricians in comparison to Cairo, the capital government.

## Supplementary Information

**Additional file 1.** The three sections of the questionnaire addressing the economic and emotional impact of COVID-19 pandemic on phoniatricians’ practice in Egypt.

## Data Availability

The datasets used and analyzed during the current study are available from the corresponding author on reasonable request.

## Abbreviations

COVID-19: Coronavirus disease of 2019
ESPL: Egyptian Society of Phoniatricians & Logopedics
SLTs: Speech and language therapists
PPE: Personal protective equipment
SARS: Severe acute respiratory syndrome
