# Supplementary material for: Economic and emotional impact of COVID-19 pandemic on phoniatricians’ practice in Egypt
Source: Egypt J Otolaryngol. 2022 Feb 14;38(1):25. doi: 10.1186/s43163-022-00217-x (PMC8852922; doi:10.1186/s43163-022-00217-x)
Supplement: Supplementary file 1 — Additional file 1. The three sections of the questionnaire addressing the economic and emotional impact of COVID-19 pandemic on phoniatricians’ practice in Egypt. [file 43163_2022_217_MOESM1_ESM.docx]

**Appendix:**

**The three sections of the questionnaire addressing the Economic and Emotional impact of Covid 19 Pandemic on Phoniatricians' practice in Egypt:**

| Section I : Socio- demographic data | Choices |
| --- | --- |
| Age | - 20-30 - 31-40 - 41-50 - Above 50 |
| Sex | - Male - Female |
| Marital Status | - Single - Married - Married & have children |
| Family Size | - 2 - 3 - 4 - More than 4 |
| Length of professional experience | - Less than 5 years - 5-10 - 11-15 - 16-20 - More than 20 |
| Location of practice | - Cairo - Alexandria - Delta - Upper Egypt - Other |
| Professional setting | - Partner/ owner of a private clinic. - Employed in Private practice - Employed in public structure - Have public & private practice |
| History of any emotional problem | - No - Anxiety - Fear - Depression - Others |

| Section II : Economical Section data | Choices |
| --- | --- |
| Q9  Weekly average working time before the pandemic (Mid- March till present time) | - More than 25 hours - 20-25 hours - 10-19 hours - Less than 10 hours - Not working |
| Q10  Weekly average working time during the pandemic (Mid- March till present time) | - More than 25 hours - 20-25 hours - 10-19 hours - Less than 10 hours - Not working |
| Q11  The likelihood of the negative effect of the pandemic on practice | - A lot - a little - Not at all |
| Q12  Continuation of practice during Covid 19 Pandemic | - Continued the same - Closed - Reduced to 50 percent of its power - Reduced to urgent procedures/ consultations |
| Q13  The relation between expenses & income during Covid 19 pandemic | - I keep the balance between my income & expenses - My expenses exceed my income - My income exceeds my expenses |
| Q14  The cause of increasing of expenses burden in the Covid 19 Pandemic | - Protective personal equipment financial burden - Internet usage - Family issues - Decreased income - All of the above - None of the above |
| Q15  Availability of PPEs | - Available - Not available |
| Q16  Providing PPEs | - Provided by your organization - You have to buy them at your expenses |
| Q17  The effect of the availability of the PPEs on clinical practice | - Yes - No - Sometimes |
| Q18  The price of the personal protective equipment during the Pandemic | - Is the same - Has increased to a large extent |
| Q19  PPEs used before the pandemic | - Gloves, masks, white coats - Gloves, masks, gowns - Gloves, masks, face shield & disposable gowns |
| Q20  The time of complete or partial closure of work | - Just when the danger of the pandemic was felt - After the national quarantine was declared |
| Q21  Patients understanding of the motives beyond the closure or reduction | - Yes - No - Not all of them |
| Q22  Way of conduction of consultations for urgent conditions | - Through telephone - Online consultation - Face to face - More than one of the above - All of the above |
| Q23  The main source of financial income during the Covid 19 Pandemic | - Online sessions - Governmental salary - Private Practice - Other practices than Phoniatrics - More than one of the above |
| Q24  Thinking about plan b & c to cover finances during the pandemic | - Yes - No - Did not find any idea to help |
| Q25  Thinking about career shift | - Yes - No |
| Q26  Regaining of full practice after the national reopening in case of partial or complete closure of work | - Yes - No |
| Q27  Safety measures from infection | - Body temperature measurement - Asking about symptoms - Usage of disinfectants - Reducing number of cases - Providing cases with masks & gloves - More than one of the above - All of the above - None of the above |
| Q28  The likelihood of getting infection during phoniatric practice | - Yes - No - May be |
| Q29  Getting infected with coronavirus | - Yes, confirmed case - Suspected case - No |
| Q30  Place of remediation from Covid 19 | - Home (isolation) - Hospital - Did not need |
| Q31  Covering the financial issues | - Medical insurance - Own budget |
| Q32  Knowing colleagues in the field of Phoniatrics who were Covid 19 positive | - Yes - No |
| Q33  Emotional effect of the pandemic | - Scared - Frustrated - Anxious - Concerned - None of the above |
| Q34  The pandemic effect on decision about continuing practice during the pandemic | - Yes - No |
| Q35  The degree of the effect of the consequences of the Covid 19 Pandemic | - Major degree - Moderate degree - Minor degree |
| Q36  The degree the pandemic will continue to affect daily practice | - Major degree - Moderate degree - Minor degree |
| Q37  The burden causes during the pandemic | - Being in quarantine - Children care - Health & being sick with coronavirus - Financial issues - Uncertainty about job - Increasing conflicts with people around - More than one of the above - All of the above |
| Q38  Spending time during quarantine | - The same routine as before - Care of family - Developing professionally - Part time work - Reading & searching about the Corona news - More than one of the above |

| Section III:  Emotional Section Data | Choices |
| --- | --- |
| Q39  Degree of worry from getting infection/reinfection during practice | - A lot - A little - Not at all |
| Q40  Type of feeling when thinking about Covid 19 Pandemic | - Sad - Angry - Concerned - Scared - Anxious - None of the above |
| Q41  The likelihood of increasing stress level as a result of the pandemic | - To a great extent - To a moderate extent - To a little extent |
| Q42  The degree of the emotional effect of professional & economical problem | - To a great extent - To a moderate extent - To a little extent |
| Q43  The likelihood of the effect of professional & economical problem on relations with colleagues at work | - Yes - No |
| Q44  The likelihood of professional & economical problem on productivity at work | - Yes - No |
| Q45  The Likelihood of effect of professional & economical problem on relations with family and friends | - Yes - No |
| Q46  The need to take medications for anxiety or depression | - Yes - No |
| Q47  The need of psychological support | - Yes - No |
| Q48  Degree of worry about professional future | - A lot - A little - Not at all |
| Q49  Most causes of worry | - The pandemic - Corona will affect the clients financially& economically - Chance of losing job or being laid off by employer - Other |
